# Comparison of Classic Laryngeal Mask Airway and Endotracheal Intubation for Airway Management During Short Urologic Procedures in the Prone Position: A Randomized Controlled Trial

**DOI:** 10.1155/anrp/1390565

**Published:** 2026-10-25

**Authors:** Naeimeh Naeimi Bafghi, Maryam Shamsoddini Motlagh, Neda Naeimi Bafghi, Vida Mirzaie

**Affiliations:** ^1^ Clinical Research Center Shahid Bahonar Hospital Kerman University of Medical Sciences Kerman Iran kmu.ac.ir; ^2^ Department of Anatomical Science Afzalipour School of Medicine Kerman University of Medical Sciences Kerman Iran kmu.ac.ir

**Keywords:** airway management, endotracheal tube, laryngeal mask airway, prone position, urologic surgery

## Abstract

**Background:**

Airway management in the prone position remains a major challenge in anesthesia, particularly during short urologic procedures. Although endotracheal intubation (ETT) is considered the standard technique, the Classic laryngeal mask airway (LMA) has emerged as a less invasive alternative in patients without an anticipated difficult airway. This study aimed to compare the efficacy and safety of Classic LMA versus ETT for airway management in prone‐position urologic surgeries.

**Methods:**

In this randomized clinical trial, 70 patients undergoing prone‐position urologic surgery with an operative duration of ≤ 90 min were assigned to either the Classic LMA group or ETT group (*n* = 35 each). Primary outcomes included intraoperative ventilatory performance assessed by peak airway pressure (Ppeak), need for conversion of airway technique, and airway‐related complications. Secondary outcomes included intraoperative hemodynamic and respiratory parameters, including heart rate (HR), systolic and diastolic blood pressure (SBP/DBP), oxygen saturation (SpO_2_), end‐tidal carbon dioxide (ETCO_2_), Ppeak, minute ventilation (VE), and postoperative complications.

**Results:**

Baseline demographic and perioperative characteristics were comparable between groups. No significant differences were observed between the Classic LMA and ETT groups regarding intraoperative hemodynamic variables, SpO_2_, ETCO_2_, or VE. Ppeak was significantly higher in the ETT group (*p* < 0.001). During recovery, significant time‐related changes were observed in hemodynamic and respiratory parameters in both groups, although no clinically significant intergroup differences were identified. Postoperative complications, including sore throat and laryngospasm, were infrequent and comparable between groups.

**Conclusion:**

Classic LMA provided airway maintenance and ventilation comparable to ETT in short prone‐position urologic surgeries and may be considered as a feasible airway device in patients meeting appropriate airway assessment criteria.

**Trial Registration:** Iranian Registry of Clinical Trials (IRCT): IRCT20250729066688N1

## 1. Introduction

Airway management in the prone position remains one of the most challenging aspects of anesthetic practice. Although the prone position provides excellent surgical exposure and is routinely used for spinal, lumbar, and urologic procedures, it substantially limits access to the airway once the patient has been positioned. Consequently, airway‐related complications such as accidental extubation, device displacement, airway obstruction, or the need for emergency airway intervention become considerably more difficult to manage than in the supine position. Furthermore, impaired secretion drainage and the potential risk of aspiration may increase perioperative respiratory complications, highlighting the importance of selecting a secure and reliable airway management technique for prone‐position surgery [1–4]. Endotracheal intubation (ETT) is generally regarded as the standard method for airway management during prone‐position surgery because it provides a definitive airway with effective protection against aspiration. Nevertheless, tracheal intubation requires direct laryngoscopy and greater technical expertise and is associated with sympathetic stimulation, postoperative sore throat, cough, dental trauma, and pharyngeal tissue injury [5, 6]. In contrast, the laryngeal mask airway (LMA), a supraglottic airway device, has become an attractive alternative because of its ease of insertion, reduced airway stimulation, lower incidence of postoperative airway discomfort, and ability to facilitate either spontaneous or controlled ventilation (VE) [7–10].

Despite these advantages, concerns remain regarding its ability to maintain an adequate airway seal and protect against aspiration during procedures performed in the prone position [11]. The use of supraglottic airway devices in prone‐position anesthesia remains controversial. While these devices are widely accepted as rescue tools in cases of accidental extubation, their use as a primary airway management technique in elective prone surgeries is still debated [7, 12–14]. Several clinical studies have demonstrated encouraging results. Mahmoud and Rashwan reported comparable airway success rates between LMA and ETT in pediatric patients undergoing prone surgery, with fewer perioperative complications in the LMA group [2]. Similarly, Ng et al. showed that LMA could be safely used for induction and maintenance of anesthesia in selected prone‐position ambulatory procedures when performed by experienced anesthesiologists [5]. Furthermore, a systematic review by Abrishami et al., including 12 studies and 526 patients, reported successful supraglottic airway placement in 87.5%–100% of cases and adequate VE in 83.3%–100%, supporting the feasibility of LMA in carefully selected patients [15]. Likewise, Gable et al. reviewed more than 1000 reported cases of LMA use in the prone position and found that most complications were minor, primarily consisting of mask displacement, laryngospasm, and the need for assisted VE. However, they emphasized that successful use of LMA depends on strict patient selection, the availability of an established airway rescue strategy, and the ability to promptly reposition patients to the supine position if airway compromise occurs [16]. Despite these favorable findings, concerns regarding patient safety persist. Staender argued that the limited reduction in anesthesia induction time reported by Olsen et al. did not justify a possible reduction in airway safety margins. Drawing on principles of resilience engineering, he suggested that routine use of LMA in the prone position could represent a “migration of safety boundaries” and a “normalization of deviance,” emphasizing that the absence of catastrophic complications should not be interpreted as evidence of safety. He therefore cautioned against compromising the principle of primum non nocere (“first, do no harm”) for relatively small gains in operating room efficiency [17, 18].

Overall, current evidence suggests that LMA may be a feasible alternative to ETT in selected prone‐position procedures; however, the available literature is limited by relatively small sample sizes, heterogeneous surgical populations, and variations in airway devices and anesthetic techniques. Moreover, few randomized clinical trials have directly compared LMA with ETT in adult patients undergoing short‐duration urologic surgery in the prone position. Therefore, this randomized clinical trial was designed to compare intraoperative ventilatory performance (assessed by peak airway pressure [Ppeak]), the rate of conversion to an alternative airway technique, and airway‐related complications between the Classic LMA and ETT in patients undergoing prone‐position urologic procedures lasting ≤ 90 min.

### 1.1. Research Question and Hypothesis

The primary research question of this study was whether the Classic LMA provides airway management performance comparable to oral ETT in adult patients undergoing short‐duration (≤ 90 min) urologic surgery in the prone position.

We hypothesized that, in appropriately selected patients (see Section 2.2 for eligibility criteria), the Classic LMA would provide comparable VE, oxygenation, and hemodynamic stability to oral ETT, without increasing airway‐related complications.

## 2. Materials and Methods

### 2.1. Study Design and Ethical Considerations

The study was carried out in the Urology Operating Room of Kerman University of Medical Sciences at Shahid Bahonar Hospital, Iran, in 2025, following approval by the institutional ethics committee and registration in the Iranian Registry of Clinical Trials (ethical approval code IR.KMU.AH.REC.1404.114). Written informed consent was obtained from all participants prior to enrollment. This randomized clinical trial was designed, conducted, and reported in accordance with the Consolidated Standards of Reporting Trials (CONSORT) 2010 guidelines (Figure 1).

**FIGURE 1:**
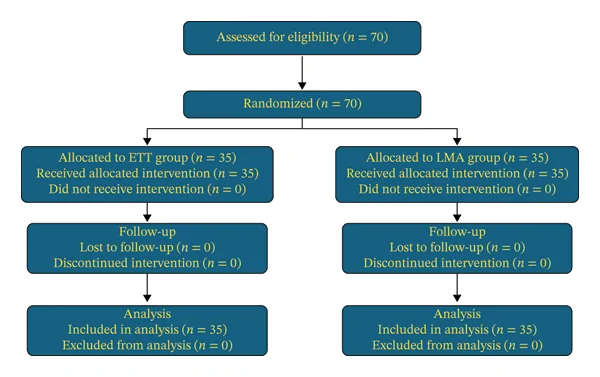
CONSORT flow diagram of participant recruitment, randomization, allocation, follow‐up, and analysis.

### 2.2. Participants

The study population consisted of 70 patients aged 18–65 years of both sexes who were scheduled for elective urologic surgery under general anesthesia in the prone position with an expected duration of ≤ 90 min. Preoperative airway assessment was performed by an experienced anesthesiologist and included evaluation of the Mallampati classification, thyromental distance, mouth opening, and neck mobility. An anticipated difficult airway was defined as a Mallampati class III or IV, a thyromental distance < 6 cm, a limited mouth opening (< 3 cm), or restricted neck extension. Patients meeting any of these criteria were excluded from the study. Inclusion was restricted to ASA physical status I–II patients to minimize the confounding effect of underlying cardiopulmonary comorbidities on hemodynamic and respiratory outcomes. The majority of procedures were percutaneous nephrolithotomy (PCNL; 66/70 patients), while four patients underwent percutaneous nephrostomy requiring general anesthesia. All four nephrostomy procedures were performed in the Classic LMA group (4/35), while all 35 patients in the ETT group underwent PCNL.

### 2.3. Exclusion Criteria

Exclusion criteria were evaluated during preoperative screening, before randomization, and included any condition that would limit the use of an LMA. An anticipated need for corticosteroid administration before or during anesthesia was also considered an exclusion criterion, given its potential effect on airway inflammatory response and postoperative airway complications [19]. A serious intraoperative complication requiring a change in the VE technique was predefined as persistent SpO_2_ < 90% despite repositioning, Ppeak > 40 cmH_2_O, or inability to achieve adequate VE as confirmed by capnography. Since none of these events occurred during the study, no patient required conversion of the airway technique or exclusion after randomization (Figure 1).

Additional exclusion criteria included:

• History of allergy to any study medications or airway devices
• Obesity (BMI > 30 kg/m^2^)
• Nonfasting status
• Emergency surgery
• Respiratory diseases such as asthma or chronic obstructive pulmonary disease (COPD)
• Use of corticosteroids before or during anesthesia
• Development of serious intraoperative complications requiring alteration of the VE technique
• Surgical duration exceeding 90 min

### 2.4. Randomization and Blinding

After obtaining written informed consent, eligible patients were randomly assigned into two equal groups (*n* = 35 each) using a random number table and the RAND function generated in Microsoft Excel. Allocation concealment was ensured using sequentially numbered, opaque, and sealed envelopes. A double‐blind design was implemented in which both the patient and the investigator responsible for compiling and analyzing the collected outcome data were unaware of the assigned airway device. Intraoperative data were recorded by a member of the anesthesia team with access to the anesthesia record, who was therefore not blinded to group allocation. However, the investigator who subsequently organized and analyzed the outcome data had access only to the standardized data collection form, on which the airway device was coded as Group A or Group B.

• Group A: Classic LMA group
• Group B: ETT group

### 2.5. Ventilator Settings and Intraoperative Monitoring

Mechanical VE settings were standardized in both groups with a tidal volume of 6 mL/kg and a respiratory rate of 14 breaths/minute. End‐tidal carbon dioxide (ETCO_2_) was continuously monitored using capnography, and the respiratory rate was adjusted if ETCO_2_ exceeded 40 mmHg.

All patients received standard intraoperative monitoring, hydration, and anesthetic management according to reference anesthesia guidelines. Vital signs were recorded every 15 min during surgery, upon arrival to the recovery room, and 30 min after admission to recovery.

### 2.6. Data Collection

Baseline demographic and clinical characteristics were collected using a standardized data collection form. Recorded variables included anesthesia‐related complications, airway pressure, respiratory and ventilatory indices (expired tidal volume, arterial oxygen saturation, and ETCO_2_), duration of surgery, and intraoperative hemodynamic parameters.

### 2.7. Sample Size Calculation

The primary outcome of this trial was Ppeak, a continuous variable reflecting intraoperative ventilatory performance. Sample size was calculated manually using the standard formula for comparison of two independent means: *n* = [2 × (*Z*_*α*/2 + *Z*_*β*)^2^ × *σ* ^2^]/Δ^2^ assuming a minimum clinically important difference (Δ) of 3 cm H_2_O in Ppeak between the Classic LMA and ETT groups, with a pooled standard deviation (SD) (*σ*) of 4.5 cm H_2_O, derived from comparative data on Ppeak between LMA and ETT reported by Malekshoar et al. [20]. With a 95% confidence level (*Z*_*α*/2 = 1.96) and 80% statistical power (*Z*_*β* = 0.84), the calculated sample size was 35 patients per group. Therefore, a total of 70 patients were enrolled in the study.

### 2.8. Statistical Analysis

Statistical analysis was performed using IBM SPSS Statistics 20. The normality of continuous variables was assessed using the Shapiro‐Wilk test. Normally distributed continuous variables were expressed as mean ± SD and compared between groups using the independent‐samples *t*‐test; nonnormally distributed variables were expressed as median (interquartile range) and compared using the Mann–Whitney *U* test. Categorical variables were presented as frequency (percentage) and compared using the Chi‐square test or Fisher’s exact test, as appropriate.

Repeated intraoperative measurements of hemodynamic variables and respiratory variables were analyzed using a linear mixed‐effects model, with group (Classic LMA vs. ETT), time, and the group × time interaction included as fixed effects, and patient included as a random effect to account for within‐subject correlation across repeated measurements. A *p* value < 0.05 was considered statistically significant for all analyses.

## 3. Results

### 3.1. Baseline Characteristics

No significant differences were observed between the ETT and Classic LMA groups regarding demographic characteristics, baseline hemodynamic variables, or operative duration (all *p* > 0.05). The two groups were therefore considered comparable at baseline (Table 1).

**TABLE 1:** Baseline characteristics and preoperative variables.

Variable	ETT (*n* = 35)	LMA (*n* = 35)	*p* value
Age (yr)	46.68 ± 14.48	48.28 ± 14.27	0.613
BMI (kg/m^2^)	26.42 ± 3.15	24.20 ± 2.85	0.516
Surgery duration (min)	68.57 ± 12.75	70.71 ± 13.88	0.502
Male/Female	17/18	18/17	0.811
ASA I/II	20/15	20/15	0.99
Baseline SBP	142.31 ± 30.29	138.26 ± 26.91	0.264
Baseline DBP	87.23 ± 13.14	91.17 ± 13.02	0.217
Baseline HR	82.65 ± 16.39	80.37 ± 19.03	0.920
Baseline SpO_2_	96.25 ± 1.72	95.97 ± 1.54	0.477

### 3.2. Intraoperative Hemodynamic Parameters

Mixed‐model analysis demonstrated significant time‐dependent changes in heart rate, systolic blood pressure, and diastolic blood pressure during surgery (*p* < 0.05 for all). However, no significant overall group effect was observed between the ETT and Classic LMA groups. Additionally, the interaction between time and group was not statistically significant for these variables, indicating that the pattern of hemodynamic changes over time was generally similar in both groups.

Heart rate values tended to remain slightly lower in the ETT group throughout most intraoperative measurements. Similarly, systolic and diastolic blood pressure showed physiological fluctuations during surgery in both groups, particularly after positioning patients in the prone position, but without clinically meaningful intergroup differences (Table 2, Figure 2, supporting Table S1).

**TABLE 2:** Summary of mixed‐model analysis of intraoperative hemodynamic variables.

Variable	Group effect *p*	Time effect *p*	Group × time *p*	Interpretation
HR	0.132	0.013	0.060	Time‐dependent changes only
SBP	0.691	0.001	0.153	No between‐group difference
DBP	0.658	< 0.001	0.081	Time‐dependent changes

**FIGURE 2:**
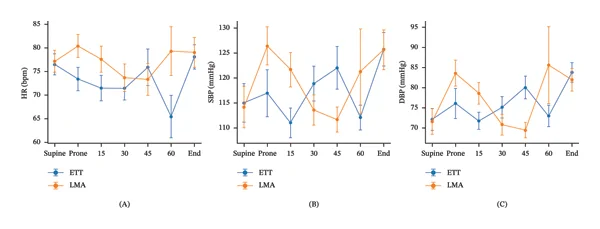
Hemodynamic changes during surgery; (A) heart rate, (B) systolic blood pressure, and (C) diastolic blood pressure; data presented by mean ± SEM.

### 3.3. Intraoperative Respiratory Parameters

Intraoperative respiratory variables remained generally stable in both groups throughout surgery. No significant differences were observed between groups regarding SpO_2_, ETCO_2_, or minute VE. SpO_2_ values remained within the normal range during all measurement periods, indicating adequate oxygenation in both airway management techniques.

ETCO_2_ demonstrated a mild but significant time‐related increase during surgery (*p* < 0.001), although the trend was comparable between groups. Minute VE remained stable without significant group or time effects.

Ppeak was the only respiratory parameter that demonstrated a significant group effect (*p* < 0.001), with consistently higher values observed in the ETT group compared with the Classic LMA group. A significant time effect was also identified for Ppeak, suggesting dynamic intraoperative changes in airway pressure during surgery (Table 3, Figure 3, supporting Table S2).

**TABLE 3:** Summary of mixed‐model analysis of intraoperative respiratory variables.

Variable	Group effect (*p* value)	Time effect (*p* value)	Group × time interaction (*p* value)	Interpretation
SpO_2_	0.715	0.627	0.460	No significant differences between groups or over time
ETCO_2_	0.323	< 0.001	0.279	Significant change over time only
Peak airway pressure (Ppeak)	< 0.001	< 0.001	0.050	Significant group and time effects; borderline interaction
Minute ventilation (VE)	0.330	0.181	0.674	No significant differences between groups or over time

**FIGURE 3:**
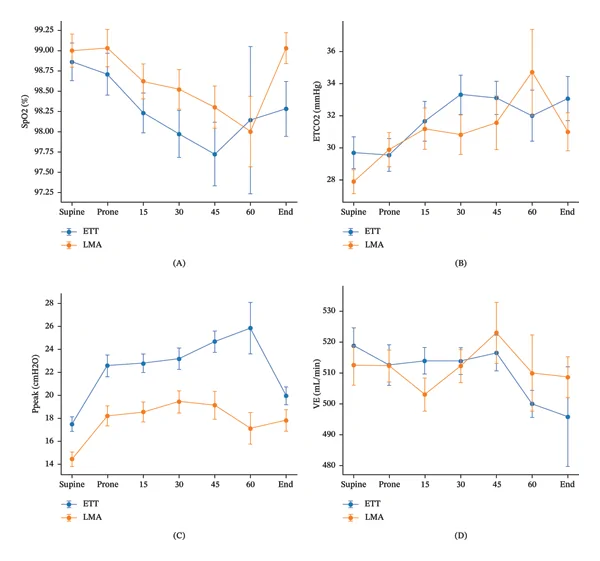
Respiratory parameters during surgery; (A) SpO_2_, (B) ETCO_2_, (C) peak, and (D) minute ventilation, data presented by mean ± SEM.

### 3.4. Recovery Hemodynamic and Respiratory Parameters

During the recovery period, systolic blood pressure, diastolic blood pressure, heart rate, and SpO_2_ all demonstrated significant changes over time. Both systolic and diastolic blood pressure decreased during the first 30 min of recovery in both groups.

A significant interaction between time and group was observed for systolic and diastolic blood pressure, indicating slight differences in the recovery trend between airway management techniques. However, no significant overall group effect was found for any recovery variable.

Heart rate decreased significantly during recovery in both groups, while SpO_2_ values increased over time and remained within normal physiological ranges. The patterns of change for heart rate and oxygen saturation were similar between groups (Table 4, Figure 4, supporting Table S3.

**TABLE 4:** Repeated‐measures analysis of recovery variables.

Variable	Group effect (*p* value)	Time effect (*p* value)	Group × time interaction (*p* value)	Interpretation
SBP	0.594	0.005	0.017	Significant time and interaction effects
DBP	0.103	< 0.001	0.042	Significant time and interaction effects
HR	0.653	< 0.001	0.980	Significant time effect only
SpO_2_	0.178	< 0.001	0.678	Significant time effect only

**FIGURE 4:**
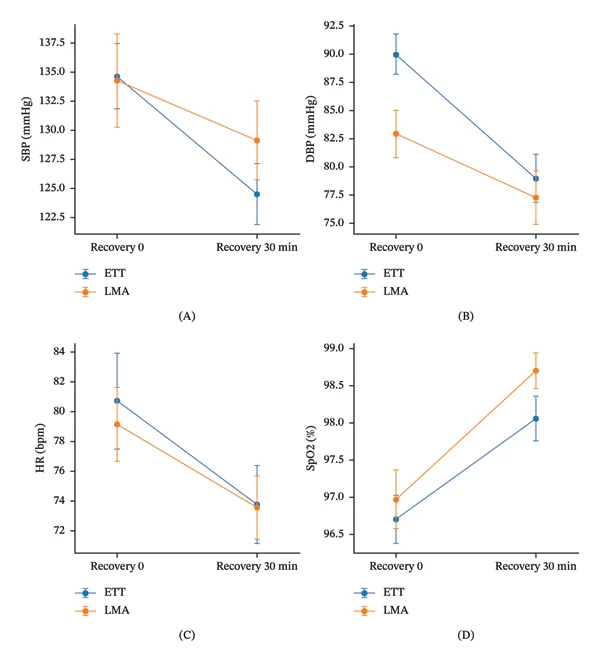
Recovery trends; (A) systolic blood pressure, (B) diastolic blood pressure, (C) heart rate, and (D) SpO_2_. Data presented by mean ± SEM.

### 3.5. Perioperative and Postoperative Complications

The incidence of postoperative complications, including sore throat, laryngospasm, nausea, and vomiting, was low in both groups. No statistically significant differences were observed between the endotracheal tube and Classic LMA groups regarding perioperative or postoperative complications (all *p* > 0.05).

One patient in the ETT group experienced inadvertent endobronchial (right mainstem) intubation, detected by a sudden rise in Ppeak and desaturation shortly after intubation and before the start of surgery. The tube was promptly withdrawn to the appropriate depth under auscultation, and the patient completed the procedure without further complications or long‐term sequelae (Table 5).

**TABLE 5:** Postoperative airway complications.

Complication	ETT *n* (%)	LMA *n* (%)	*p* value
Sore throat	3 (8.6)	1 (2.9)	0.303
Laryngospasm	0	1	0.314
Nausea/Vomiting	1	0	0.314

## 4. Discussion

The present randomized clinical trial compared Classic LMA and oral ETT for airway management in adult patients undergoing short‐duration urologic procedures in the prone position. The main findings of this study were that both airway techniques maintained adequate oxygenation and ventilation throughout surgery and were associated with comparable intraoperative hemodynamic stability. Among the respiratory variables evaluated, Ppeak was consistently lower in the Classic LMA group, while perioperative and postoperative complications were infrequent and did not differ significantly between groups. Airway management in the prone position remains a challenging aspect of anesthetic practice because access to the airway becomes limited after positioning. Consequently, the selection of an airway device must ensure adequate VE while minimizing the risk of airway‐related complications. Furthermore, although our findings support the feasibility of the Classic LMA in this setting, its use in the prone position requires careful and continuous monitoring by the anesthesiologist. Continuous assessment of oxygen saturation, ETCO_2_, airway pressure, VE, and hemodynamic status is essential to detect early complications. In addition, an appropriate airway rescue strategy and readiness for prompt airway intervention should be maintained throughout the procedure. In the present study, all surgical procedures were completed using the initially assigned airway device without requiring conversion to an alternative airway technique. Furthermore, oxygen saturation remained within normal limits throughout the procedure in both groups, indicating that both Classic LMA and ETT were capable of maintaining adequate oxygenation under the study conditions. Hemodynamic variables demonstrated significant changes over time, which likely reflected the physiological effects of anesthesia, surgical stimulation, and positional changes during the procedure. However, no significant differences were observed between the two airway management techniques regarding heart rate, systolic blood pressure, or diastolic blood pressure during surgery. These findings suggest that, under standardized anesthetic conditions, the choice of airway device may have a limited impact on overall intraoperative cardiovascular stability. Similar observations have been reported in previous investigations. Sharma et al. demonstrated stable hemodynamic responses associated with LMA use in both prone and supine positions, while Mahmoud and Rashwan reported comparable cardiovascular parameters in pediatric patients managed with either LMA or ETT during prone‐position surgery [2, 21].

During the recovery period, respiratory outcomes in the present study support the feasibility of Classic LMA for selected short‐duration prone‐position procedures. Throughout surgery, oxygen saturation and minute VE remained stable and comparable between groups. ETCO_2_ increased modestly over time in both groups; however, no significant group effect was observed. Although the ventilator protocol allowed for adjustment of respiratory rate if ETCO_2_ exceeded 40 mmHg, this threshold was not reached in any patient in either group throughout the study period (mean ETCO_2_ ranging from approximately 28–34 mmHg at all time points). Consequently, tidal volume and respiratory rate settings remained unchanged for the duration of surgery in all patients, and minute VE, calculated as the product of tidal volume and respiratory rate as displayed by the ventilator, remained stable accordingly. The modest, statistically significant time‐related rise in ETCO_2_ therefore reflects physiological changes (e.g., reduced CO_2_ clearance or altered pulmonary mechanics after prone positioning) within a clinically insignificant range, rather than a change in ventilatory settings. Importantly, the absence of significant differences between groups indicates that alveolar VE was maintained similarly with both airway devices. The most notable respiratory finding was the significantly lower Ppeak observed in the Classic LMA group. Ppeak reflects the pressure required to deliver the set tidal volume and is influenced by airway resistance and respiratory system compliance. The lower Ppeak observed with the Classic LMA may reflect differences in airway geometry and flow resistance between a supraglottic airway and an endotracheal tube. Importantly, this difference was not accompanied by differences in oxygenation, ETCO_2_, or minute VE in the present study. Although the higher Ppeak observed in the ETT group were not associated with impaired gas exchange or clinically significant complications in the present study, lower airway pressures may be advantageous in patients with reduced pulmonary compliance or limited respiratory reserve. Similar findings have been reported by Malekshoar et al., who observed significantly lower airway pressures with LMA despite comparable oxygenation and VE parameters between airway devices [20, 22].

During the recovery period, systolic blood pressure, diastolic blood pressure, heart rate, and oxygen saturation changed significantly over time in both groups. While heart rate and oxygen saturation demonstrated similar recovery patterns regardless of airway technique, systolic and diastolic blood pressure showed significant time‐by‐group interactions. Patients in the ETT group exhibited higher blood pressure values upon arrival in the recovery room followed by a more pronounced decline during the first 30 min of recovery, reflected in a significant time‐by‐group interaction. One possible explanation is greater sympathetic stimulation associated with tracheal instrumentation and extubation compared with supraglottic airway devices, a mechanism proposed in previous studies [23, 24]; however, our study did not directly measure catecholamine levels or hemodynamic responses at the precise moment of airway removal, and this interpretation should therefore be considered hypothesis‐generating rather than confirmed by our data. Importantly, no significant overall group effect was observed for blood pressure during either the intraoperative or recovery periods, indicating that any such difference was transient, modest in magnitude, and of limited clinical significance in this relatively healthy (ASA I–II) study population.

Postoperative airway‐related complications were uncommon in both groups. Although sore throat occurred more frequently in the ETT group, the difference did not reach statistical significance. Similarly, isolated cases of laryngospasm, postoperative nausea and vomiting were observed without significant between‐group differences. The low incidence of complications in both groups may reflect careful patient selection, standardized anesthetic management, and the experience of the anesthesia team. These findings are consistent with previous reports indicating that supraglottic airway devices are associated with complication rates comparable to those of ETT when used in patients meeting the eligibility criteria outlined in Section 2.2 [23, 25, 26].

The present study has several strengths. First, the randomized design minimized selection bias and ensured comparable baseline characteristics between groups. Second, repeated intraoperative measurements provided a comprehensive evaluation of hemodynamic and respiratory responses throughout surgery. Third, the study focused on a relatively homogeneous surgical population undergoing procedures of similar duration and complexity, thereby reducing potential confounding factors.

Several limitations should also be considered. This was a single‐center study with a relatively small sample size, which may have limited the ability to detect differences in uncommon adverse events. In addition, only patients with ASA physical status I–II undergoing elective urologic procedures lasting 90 min or less were included. Therefore, the findings may not be generalizable to patients with significant cardiopulmonary disease, obesity, difficult airways, or those undergoing prolonged surgical procedures. Finally, only Classic LMA was evaluated, and the results may not necessarily apply to other supraglottic airway devices.

## 5. Conclusion

In conclusion, the findings of this study indicate that Classic LMA maintained adequate VE and oxygenation and provided perioperative hemodynamic profiles comparable to those achieved with oral ETT in patients without an anticipated difficult airway undergoing short‐duration prone urologic surgery. In addition, Classic LMA was associated with lower Ppeak without an increase in perioperative complications. These findings suggest that Classic LMA may be considered a reasonable alternative to ETT in patients undergoing short‐duration procedures in the prone position. Future studies should evaluate the safety and effectiveness of Classic LMA in broader and clinically distinct patient populations, including pediatric patients and adults with comorbidities such as hypertension or ischemic heart disease. Prospective studies in these populations, particularly with larger sample sizes and standardized perioperative monitoring, may help determine whether the respiratory and hemodynamic findings observed in the present study are reproducible in patients with different physiological and cardiovascular characteristics.

### 5.1. Limitations

1. The four nephrostomy procedures were unevenly distributed between groups (all occurring in the LMA group), which, despite the small number of cases, should be considered as a potential source of heterogeneity.
2. This study did not record the time required to secure the airway or hemodynamic parameters at the specific moment of device placement; a period previously associated with the greatest hemodynamic fluctuations. Vital signs were recorded at baseline, at 15‐min intervals intraoperatively, and during recovery, which may not have captured acute peri‐insertion changes. Future studies should incorporate continuous or high‐frequency monitoring around the time of airway placement to better characterize this period.

## Author Contributions

Naeimeh Naeimi Bafghi, Neda Naeimi Bafghi, and Maryam Shamsoddini Motlagh contributed to data collection, patient monitoring, and manuscript drafting. Naeimeh Naeimi Bafghi contributed to study design, statistical analysis, and interpretation of results. Vida Mirzaie conceived the study, supervised the project, wrote the manuscript, and is the corresponding author.

## Funding

This study was financially supported by Kerman University of Medical Sciences, a publicly funded academic institution.

## Disclosure

The funding body had no role in the design of the study, data collection, analysis, interpretation of data, or in writing the manuscript. All authors have read and approved the final manuscript.

## Ethics Statement

This study was conducted in accordance with the ethical standards and was approved by the Ethics Committee of Kerman University of Medical Sciences, Iran (Ethics approval code: IR.KMU.AH.REC.1404.114). Written informed consent was obtained from all participants prior to their inclusion in the study.

## Consent

Written informed consent was obtained from all participants prior to their inclusion in the study.

## Conflicts of Interest

The authors declare no conflicts of interest.

## Supporting Information

Additional supporting information can be found online in the Supporting Information section.

## Supporting information


**Supporting Information** Supporting Table S1 presents detailed results of the intraoperative hemodynamic variables. Supporting Table S2 presents detailed results of the intraoperative respiratory variables. Supporting Table S3 presents detailed results of the recovery variables.

## Data Availability

The datasets generated and/or analyzed during the current study are available from the corresponding author on reasonable request.

## References

[1] Kumar V. , Lalitha K. , and Lone T. , Use of Classic Laryngeal Mask Airway Inserted in Prone Position for Controlled Ventilation: A Feasibility Study, Indian Journal of Anaesthesia. (2008) 52, no. 6, 813–817.

[2] Mahmoud H. and Rashwan D. , Use of the Classic Laryngeal Mask Airway Versus an Endotracheal Tube in Children Undergoing Elective Surgery in the Prone Position: A Prospective Randomized Feasibility Study, Journal of Anesthesia & Clinical Research. (2018) 9.

[3] Weksler N. , Laryngeal Mask in Prone Position: Pure Exhibitionism or a Valid Technique, Minerva Anestesiologica. (2007) 73, no. 1-2, 33–37. 17356505

[4] Valero R. , Serrano S. , Adalia R. et al., Anesthetic Management of a Patient in Prone Position With a Drill Bit Penetrating the Spinal Canal at C1-C2, Using a Laryngeal Mask, Anesthesia & Analgesia. (2004) 98, no. 5, 1447–1450, 10.1213/01.ane.0000111102.52964.7f. 15105228

[5] Ng A. , Raitt D. G. , and Smith G. , Induction of Anesthesia and Insertion of a Laryngeal Mask Airway in the Prone Position for Minor Surgery, Anesthesia & Analgesia. (2002) 94, no. 5, 1194–1198, 10.1097/00000539-200205000-00026. 11973188

[6] Zhang K. , Supraglottic Airway Devices: A Powerful Strategy in Airway Management, American Journal of Cancer Research. (2024) 14, no. 1, 16–32, 10.62347/kjru4855. PMC10839323 38323274

[7] Stevens W. C. and Mehta P. D. , Use of the Laryngeal Mask Airway in Patients Positioned Prone for Short Surgical Cases in an Ambulatory Surgery Unit in the United States, Journal of Clinical Anesthesia. (2008) 20, no. 6, 487–488, 10.1016/j.jclinane.2008.05.009. 18929301

[8] Van Zundert T. , Brimacombe J. R. , Ferson D. Z. , Bacon D. R. , and Wilkinson D. J. , Archie Brain: Celebrating 30 Years of Development in Laryngeal Mask Airways, Anaesthesia. (2012) 67, no. 12, 1375–1385, 10.1111/anae.12003.x. 23130725

[9] Hernandez M. R. , Klock P. A. , and Ovassapian A. , Evolution of the Extraglottic Airway: A Review of Its History, Applications, and Practical Tips for Success, Anesthesia & Analgesia. (2012) 114, no. 2, 349–368, 10.1213/ane.0b013e31823b6748. 22178627

[10] Seung H. Y. and Beirne O. R. , Laryngeal Mask Airways Have a Lower Risk of Airway Complications Compared With Endotracheal Intubation: A Systematic Review, Journal of Oral and Maxillofacial Surgery. (2010) 68, no. 10, 2359–2376. 10.1016/j.joms.2010.04.017 20674126

[11] Kumar M. , Marulasidappa M. , and Parmar P. , Baska Mask®-A Third Generation Supraglottic Airway Device in Clinical Practice-A Prospective Observational Study, Indian Journal of Clinical Anaesthesia. (2020) 5, no. 4, 576–581.

[12] Ricci E. , Comparison of Endotracheal Tube (ETT) and Laryngeal Mask Airway (LMA) in Pediatric Anesthesia: A Systematic Review, Journal For International Medical Graduates. (2024) 3, no. 2.

[13] Miller V. J. and Flaherty E. E. , Comparing No-Flow Time During Endotracheal Intubation Versus Placement of a Laryngeal Mask Airway During a Simulated Cardiac Arrest Scenario, Simulation in Healthcare. (2014) 9, no. 3, 156–160, 10.1097/sih.0000000000000002. 24310162

[14] López A. , Valero R. , Hurtado P. , Gambús P. , Pons M. , and Anglada T. , Comparison of the LMA Supreme™ With the LMA Proseal™ for Airway Management in Patients Anaesthetized in Prone Position, British Journal of Anaesthesia. (2011) 107, no. 2, 265–271, 10.1093/bja/aer104. 21576096

[15] Abrishami A. , Zilberman P. , and Chung F. , Brief Review: Airway Rescue With Insertion of Laryngeal Mask Airway Devices With Patients in the Prone Position, Canadian Journal of Anesthesia/Journal canadien d’anesthésie. (2010) 57, no. 11, 1014–1020, 10.1007/s12630-010-9378-1. 20811970

[16] Gable A. , Whitaker E. , and Tobias J. , Use of the Laryngeal Mask Airway in the Prone Position, Pediatric Anesthesia and Critical Care Journal. (2015) 3, 118–123.

[17] Olsen K. S. , Petersen J. T. , Pedersen N. A. , and Rovsing L. , Self-Positioning Followed by Induction of Anaesthesia and Insertion of a Laryngeal Mask Airway Versus Endotracheal Intubation and Subsequent Positioning for Spinal Surgery in the Prone Position: A Randomised Clinical Trial, European Journal of Anaesthesiology| EJA. (2014) 31, no. 5, 259–265, 10.1097/eja.0000000000000004. 24247413

[18] Staender S. , CON: Laryngeal Masks Must Not be Used for Surgery in the Prone Position, European Journal of Anaesthesiology| EJA. (2014) 31, no. 5, 256–258, 10.1097/eja.0000000000000037. 24710088

[19] Zhang W. , Zhao G. , Li L. , and Zhao P. , Prophylactic Administration of Corticosteroids for Preventing Postoperative Complications Related to Tracheal Intubation: A Systematic Review and Meta-Analysis of 18 Randomized Controlled Trials, Clinical Drug Investigation. (2016) 36, no. 4, 255–265, 10.1007/s40261-015-0369-4. 26715108

[20] Malekshoar M. , Adibi P. , Jarineshin H. et al., Comparing the Efficiency of Laryngeal Mask Airway and Endotracheal Tube Insertion in Airway Management in Patients Planning for Elective Orthopedic Surgery Under General Anesthesia: A Randomized Clinical Trial, Bulletin of Emergency & Trauma. (2024) 12, no. 3, 111–116, 10.30476/beat.2024.102372.1509. PMC11462110 39391361

[21] Juang J. , Cordoba M. , Ciaramella A. et al., Incidence of Airway Complications Associated With Deep Extubation in Adults, BMC Anesthesiology. (2020) 20, no. 1, 10.1186/s12871-020-01191-8. PMC7597053 33121440

[22] Cherian T. R. , Laryngeal Mask Airway (LMA) and Endotracheal Intubation in Children-A Clinical Comparative Study, 2014, Rajiv Gandhi University of Health Sciences.

[23] Malekshoar M. et al., Comparison of Endotracheal Intubation and Laryngeal Mask Airway Methods Based on Dynamic Compliance and Airway Resistance, 2025.

[24] Tsuge S. , Wada A. , Iida Y. et al., Sharp Systolic Blood Pressure Elevation at Extubation is a Risk Factor for Symptomatic Epidural Hematoma After Spine Surgery, Journal of Orthopaedic Surgery. (2019) 27, no. 3, 10.1177/2309499019885449. 31802708

[25] Cooper J. R. , Use of a LMA™ and a Sequential Technique for Unanticipated Difficult Intubations, Anesthesiology. (2002) 97, no. 5, 10.1097/00000542-200211000-00050. 12411829

[26] Gong Y. , Xu X. , Wang J. , Che L. , Wang W. , and Yi J. , Laryngeal Mask Airway Reduces Incidence of Post-Operative Sore Throat After Thyroid Surgery Compared With Endotracheal Tube: A Single-Blinded Randomized Controlled Trial, BMC Anesthesiology. (2020) 20, no. 1, 10.1186/s12871-020-0932-2. PMC6961403 31937238

