# Supplementary material for: Comparison of Classic Laryngeal Mask Airway and Endotracheal Intubation for Airway Management During Short Urologic Procedures in the Prone Position: A Randomized Controlled Trial
Source: Anesthesiol Res Pract. 2026 Oct 25;2026:1390565. doi: 10.1155/anrp/1390565 (PMC13695875; doi:10.1155/anrp/1390565)
Supplement: Supplementary file 1 [file ANRP-2026-1390565-s001.docx]

# Supplementary Tables: Descriptive Statistics (Mean ± SD)

Values are mean ± SD, computed directly from the raw per-patient dataset. Sample sizes (n) vary at later intraoperative time points because procedures of shorter duration had already concluded before these time points were reached.

## Supplementary Table S1. Intraoperative hemodynamic variables (corresponds to Table 2)

| Time (min) | ETT HR (bpm) | LMA HR (bpm) | ETT SBP (mmHg) | LMA SBP (mmHg) | ETT DBP (mmHg) | LMA DBP (mmHg) |
| --- | --- | --- | --- | --- | --- | --- |
| Supine | 76.49 ± 13.15 (n=35) | 77.15 ± 13.42 (n=34) | 114.97 ± 22.99 (n=35) | 114.12 ± 24.40 (n=34) | 72.14 ± 15.94 (n=35) | 71.65 ± 18.47 (n=34) |
| Prone | 73.40 ± 14.60 (n=35) | 80.38 ± 14.24 (n=34) | 116.89 ± 27.75 (n=35) | 126.44 ± 22.37 (n=34) | 76.06 ± 22.09 (n=35) | 83.59 ± 18.78 (n=34) |
| 15 | 71.49 ± 16.00 (n=35) | 77.56 ± 16.07 (n=34) | 110.94 ± 17.71 (n=35) | 121.62 ± 20.12 (n=34) | 71.77 ± 12.57 (n=35) | 78.62 ± 15.47 (n=34) |
| 30 | 71.39 ± 13.70 (n=33) | 73.65 ± 16.23 (n=31) | 118.85 ± 20.19 (n=33) | 113.52 ± 16.93 (n=31) | 75.12 ± 15.38 (n=33) | 70.84 ± 14.23 (n=31) |
| 45 | 75.89 ± 16.43 (n=18) | 73.30 ± 15.15 (n=20) | 122.00 ± 18.17 (n=18) | 111.60 ± 11.23 (n=20) | 80.06 ± 11.91 (n=18) | 69.45 ± 8.35 (n=20) |
| 60 | 65.43 ± 11.84 (n=7) | 79.29 ± 13.65 (n=7) | 112.00 ± 6.51 (n=7) | 121.29 ± 22.57 (n=7) | 73.00 ± 7.05 (n=7) | 85.57 ± 25.24 (n=7) |
| End | 78.06 ± 14.62 (n=32) | 79.00 ± 18.20 (n=33) | 125.75 ± 19.02 (n=32) | 125.67 ± 22.91 (n=33) | 83.78 ± 13.52 (n=32) | 81.94 ± 16.13 (n=33) |

## Supplementary Table S2. Intraoperative respiratory variables (corresponds to Table 3)

| Time (min) | ETT SPO2 (%) | LMA SPO2 (%) | ETT ETCO2 (mmHg) | LMA ETCO2 (mmHg) | ETT PEAK (cmH2O) | LMA PEAK (cmH2O) | ETT VE (mL/min) | LMA VE (mL/min) |
| --- | --- | --- | --- | --- | --- | --- | --- | --- |
| Supine | 98.86 ± 1.38 (n=35) | 99.00 ± 1.18 (n=34) | 29.69 ± 5.90 (n=35) | 27.88 ± 4.38 (n=34) | 17.49 ± 3.73 (n=35) | 14.44 ± 3.65 (n=34) | 518.86 ± 34.11 (n=35) | 512.50 ± 37.58 (n=34) |
| Prone | 98.71 ± 1.53 (n=35) | 99.03 ± 1.34 (n=34) | 29.54 ± 6.01 (n=35) | 29.88 ± 6.18 (n=34) | 22.57 ± 5.54 (n=35) | 18.21 ± 5.23 (n=34) | 512.57 ± 38.91 (n=35) | 512.24 ± 29.92 (n=34) |
| 15 | 98.23 ± 1.46 (n=35) | 98.62 ± 1.26 (n=34) | 31.66 ± 7.33 (n=35) | 31.18 ± 7.48 (n=34) | 22.80 ± 4.73 (n=35) | 18.56 ± 5.06 (n=34) | 514.00 ± 25.34 (n=35) | 503.03 ± 31.32 (n=34) |
| 30 | 97.97 ± 1.67 (n=33) | 98.52 ± 1.36 (n=31) | 33.30 ± 7.06 (n=33) | 30.81 ± 6.81 (n=31) | 23.18 ± 5.29 (n=33) | 19.45 ± 5.27 (n=31) | 513.87 ± 24.04 (n=31) | 512.19 ± 29.96 (n=31) |
| 45 | 97.72 ± 1.67 (n=18) | 98.30 ± 1.17 (n=20) | 33.11 ± 4.39 (n=18) | 31.55 ± 7.50 (n=20) | 24.67 ± 3.94 (n=18) | 19.15 ± 5.40 (n=20) | 516.47 ± 23.70 (n=17) | 523.00 ± 44.38 (n=20) |
| 60 | 98.14 ± 2.41 (n=7) | 98.00 ± 1.15 (n=7) | 32.00 ± 4.20 (n=7) | 34.71 ± 7.02 (n=7) | 25.86 ± 5.93 (n=7) | 17.14 ± 3.63 (n=7) | 500.00 ± 11.55 (n=7) | 510.00 ± 32.66 (n=7) |
| End | 98.28 ± 1.92 (n=32) | 99.03 ± 1.10 (n=33) | 33.06 ± 7.69 (n=31) | 31.00 ± 6.82 (n=33) | 19.97 ± 4.31 (n=31) | 17.82 ± 5.41 (n=33) | 495.80 ± 88.20 (n=30) | 508.64 ± 38.02 (n=33) |

## Supplementary Table S3. Recovery variables (corresponds to Table 4)

| Time | ETT SBP (mmHg) | LMA SBP (mmHg) | ETT DBP (mmHg) | LMA DBP (mmHg) | ETT HR (bpm) | LMA HR (bpm) | ETT SPO2 (%) | LMA SPO2 (%) |
| --- | --- | --- | --- | --- | --- | --- | --- | --- |
| Recovery 0 | 134.64 ± 16.17 (n=33) | 134.27 ± 23.02 (n=33) | 89.97 ± 10.35 (n=33) | 82.91 ± 12.15 (n=33) | 80.70 ± 18.47 (n=33) | 79.15 ± 14.16 (n=33) | 96.70 ± 1.86 (n=33) | 96.97 ± 2.22 (n=32) |
| Recovery 30 min | 124.52 ± 14.99 (n=33) | 129.12 ± 19.52 (n=33) | 78.97 ± 12.22 (n=33) | 77.24 ± 13.61 (n=33) | 73.76 ± 15.04 (n=33) | 73.55 ± 12.14 (n=33) | 98.06 ± 1.70 (n=32) | 98.70 ± 1.36 (n=33) |
